# Overexpression of Periostin in Stroma Positively Associated with Aggressive Prostate Cancer

**DOI:** 10.1371/journal.pone.0121502

**Published:** 2015-03-17

**Authors:** Yuan Tian, Caitlin H. Choi, Qing Kay Li, Farah B. Rahmatpanah, Xin Chen, Sara Ruth Kim, Robert Veltri, David Chia, Zhen Zhang, Dan Mercola, Hui Zhang

**Affiliations:** 1 Department of Pathology, Johns Hopkins University, Baltimore, Maryland, United States of America; 2 Department of Pathology, University of California at Irvine, Irvine, California, United States of America; 3 Department of Pathology and Laboratory Medicine, University of California Los Angeles, Los Angeles, California, United States of America; Weizmann Institute of Science, ISRAEL

## Abstract

**Background:**

Periostin is an important extracellular matrix protein involved in cell development and adhesion. Previously, we identified periostin to be up-regulated in aggressive prostate cancer (CaP) using quantitative glycoproteomics and mass spectrometry. The expression of periostin was further evaluated in primary radical prostatectomy (RP) prostate tumors and adjacent non-tumorous prostate tissues using immunohistochemistry (IHC). Our IHC results revealed a low background periostin levels in the adjacent non-tumorous prostate tissues, but overexpressed periostin levels in the peritumoral stroma of primary CaP tumors.

**Methods:**

In this study, periostin expression in CaP was further examined on multiple tissue microarrays (TMAs), which were conducted in four laboratories. To achieve consistent staining, all TMAs were stained with same protocol and scored by same image computation tool to determine the total periostin staining intensities. The TMAs were further scored by pathologists to characterize the stromal staining and epithelial staining.

**Results:**

The periostin staining was observed mainly in peritumoral stromal cells and in some cases in tumor epithelial cells though the stronger staining was found in peritumoral stromal cells. Both periostin stromal staining and epithelial staining can differentiate BPH from CaP including low grade CaP (Gleason score ≤6), with significant p-value of 2.2e-16 and 0.001, respectively. Periostin epithelial staining differentiated PIN from low grade CaP (Gleason score ≤6) (p=0.001), while periostin stromal staining differentiated low grade Cap (Gleason score ≤6) from high grade Cap (Gleason score ≤6) (p=1.7e-05). In addition, a positive correlation between total periostin staining and Gleason score was observed (r=0.87, p=0.002).

**Conclusions:**

The results showed that periostin staining was positively correlated with increasing Gleason score and the aggressiveness of prostate disease.

## Introduction

In our previous effort to identify the protein changes between aggressive and non-aggressive prostate cancer (CaP), our group analyzed glycopeptides isolated from aggressive and non-aggressive prostate tumors by quantitative glycoproteomics using iTRAQ labeling of glycosite-containing peptides and tandem mass spectrometry [1]. We identified the overexpression of three glycoproteins in aggressive CaP tissues compared to non-aggressive CaP tissues. Two proteins, cathepsin L and periostin, are extracellular matrix (ECM) proteins. Cathepsin L acts as an endopeptidase, which can degrade many intracellular and extracellular proteins to modify their function. The other protein, periostin, was further verified using immunoblotting and immunohistochemistry (IHC) analyses[1]. Therefore, our observations support an important role for the tumor microenvironment in CaP progression.

Periostin, also known as osteoblast-specific factor 2(OSF-2), is an evolutionarily conserved ECM protein and a member of the fasciclin family[2]. It has been shown that periostin interacts with other ECM proteins, such as fibronectin, collagen V, and tenascin-C. Periostin also interacts with various cell-surface receptors, most notably integrins, and signals mainly via the PI3-K/Akt and other pathways to promote cell growth, cell survival, epithelial—mesenchymal transition (EMT), invasion, tumor angiogenesis and metastasis [2–5]. Recent studies have revealed that periostin is involved in the development of various tumors, such as breast, colon, lung, ovarian cancer, and prostate cancer [6–11].

In this study, we aim to verify the correlation of periostin expression and CaP aggressiveness. IHC using periostin specific antibody was performed to assess nine TMAs from multiple laboratories, which consist of a total of 3048 radial prostatectomy (RP) cores. To achieve consistent staining, the protocols and reagents were either sent to collaborator labs or TMAs were sent to our lab for TMA staining. To eliminate scoring variation from individual labs, all stained TMAs were scanned using same scanner with the same settings, and the scanned images were scored by same image computation tool to determine the total periostin staining intensities. The TMAs were further scored by pathologists to characterize the stromal staining and epithelial staining.

## Materials and Methods

### Materials

Rabbit antihuman periostin antibody were from Abcam (ab14041, Cambridge, U.K.); LSAB+ System-AP kit from Dako (Carpinteria, CA); and all other chemicals were purchased from Sigma-Aldrich (St. Louis, MO).

### Tissue microarray (TMAs)

#### TMAs from University of California Los Angeles (UCLA)

Three TMAs consist of 197 cores of non-tumorous prostate (NT), 140 cores of benign prostatic hyperplasia (BPH), 64 cores of prostate intraepithelial neoplasia (PIN), and 963 cores of prostate adenocarcinoma from 246 cases (3 cores on average for each case) were reported previously[12, 13]. NT was defined as adjacent non-cancerous prostate tissue.

#### TMAs from University of California at Irvine (UCI)

Tissue Microarray (TMA) of 480 cores consist of 199 tumor cases (with more than 10 years of follow up), 108 paired cores of prostate intraepithelial neoplasias (PIN), 7 paired cores of stroma, 3 cores of BPH, and 86 cores of calibrators and cell lines as previously reported [14, 15].

#### TMAs from Johns Hopkins University (JHU) (Bob Veltri’s lab)

Two prostate adenocarcinoma tissue microarrays (0.6 mm in diameter, 6 cores per case) referred to as TMA 681–682, were created under a CEVC EDRN grant (P.I. Alan W. Partin) constructed at Johns Hopkins University department of pathology using surgical RP resected specimens retrieved from the department of pathology archives. Each of two TMA slides represents 40 cases of CaP (4 cores per case) and tumor-matched non-cancerous prostate tissues (4 cores per case). The formalin-fixed and paraffin-embedded prostate tumors and tumor-matched non-cancerous tissues for TMA 681–682 were obtained based on application made to the Department of Pathology at Johns Hopkins Hospital through the Prostate Cancer Biorepository Network (PCBN). The use of TMAs including TMAs 681–682 to study biomarkers was approved by Johns Hopkins University IRB (Institutional Review Board) for prostate cancer.

#### TMA from Johns Hopkins University (JHU) (Hui Zhang’s lab)

The CaP tissue microarray (0.6 mm in diameter, 6 cores per case) was constructed at Johns Hopkins University using surgical RP resected specimens retrieved from the department of pathology archive at the Johns Hopkins Hospital. The use of clinical information and tumor tissue was approved by the Institutional Review Board (IRB) at Johns Hopkins University. Represented on the TMA were 60 cases of prostate adenocarcinomas (4 cores per case) and tumor-matched non-cancerous prostate tissues (2 cores per case). In addition, 60 cases of non-prostate non-cancerous control tissues were included on the microarray, consisting of normal kidney, stomach, small bowel, colon, liver, pancreas, endometrium, brain and lymph node.

### Immunohistochemistry (IHC)

The TMAs were baked at 60°C for 1 hour, deparaffinized and rehydrated. The TMAs were incubated with 5% BSA/PBS at room temperature for 45 minutes, and then incubated with 3% H_2_O_2_ at room temperature for 15 minutes prior to applying primary antibody. Rabbit antihuman periostin (Abcam, ab14041) were used in a dilution of 1:4000 to stain TMAs and were detected using the Dako LSAB+System AP kit according to the manufacturer’s instructions.

To analyze the total periostin staining, the positive pixel count (PPC, v11) algorithm (Aperio Image Scope) was applied to individual cores from 9 TMAs to compute the average pixel intensity (in a colorimetric channel corresponding to the brown IHC precipitate) of all pixels within each annotation region. Briefly, we used PPC input parameters that are defaulted for brown color quantification and then ran the algorithm. A pseudo-color “mark-up” image was generated as an algorithm result, confirming that specified inputs measured the desired color and intensity ranges. Positive stain color intensity was classified into three color coded ranges: 1) intensity weak positive (Iwp) = yellow, 2) intensity positive (Ip) = orange, and 3) intensity strong positive (Isp) = red. Percentage of staining was calculated as total number of positive pixels divided by total number of pixels and correlated with Gleason scores.

Immunostaining was also evaluated blindly by Johns Hopkins Hospital (JHH) pathologists to characterize stromal and epithelial staining localization. The TMA was scored semi-quantitatively using a four tier system based on the intensity and distribution: 0, undetectable; 1+, weak staining; 2+, medium staining, 3+, strong staining. All TMAs were scanned at 20X and IHC images were photographed using Aperio Image Scope (v11.2.0.780).

### Statistical Analysis

The Wilcoxon signed rank order test (paired, two-sided) was performed for the periostin staining in the peritumoral stroma and non-tumorous stroma which was scored by pathologists. The difference of periostin staining in either stroma or epithelial cells among different conditions of prostate disease was assessed for individual score using Chi-squared test. Pearson correlation coefficient test was used to analyze the correlation of Gleason score and the positivity of total periostin staining including both stromal and epithelial cell staining from computational scoring. *P*-values <0.05 were considered statistically significant.

## Results

Prior glycoproteomics analysis of aggressive and non-aggressive CaP tissues showed that periostin was significantly increased in aggressive prostate tumors[1]. The initial discovery was verified using IHC[1]. This study aims to verify that periostin overexpression in PCa using large sample sets from multi-laboratories and universal storing methods. The TMAs include one from Hui Zhang lab (JHU) consisting of 360 cores (60 cases), two from Bob Veltri Brady Urological Institute lab (JHU) consisting of 640 cores (80 cases), three from David Chia lab (UCLA) consisting 1364 cores (246 cases), and three from Dan Mercola lab (UCI) consisting 684 cores (199 cases).

**Fig 1:**
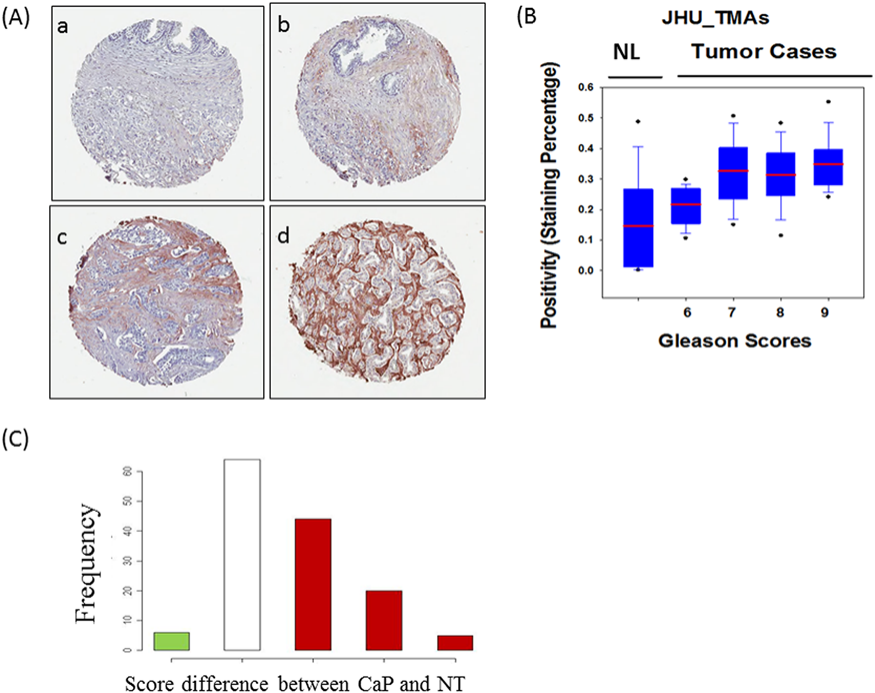
The periostin staining in prostate cancer and paired non-tumorous prostate tissues (TMA from JHU, 140 cases). (A) The pathologists’ criteria for IHC scoring.(a) 0, undetectable; (b)1+, weak staining; (c) 2+, medium staining; (d) 3+, strong staining. Periostin was mainly stained in peritumoral stroma.(B) Box plots of the positivity of periostin total staining in patient-matched prostate adenocarcinoma and non-tumorous prostate tissues. (C) Distribution of the changes in IHC scores between the peritumoral stroma and stroma from non-tumor areas (NT). On the x-axis-1: IHC score in NT is higher than in the paired cancer by 1; 0: IHC scores are the same for the paired NT and cancer; 1: IHC score in NT is lower than in the paired cancer by 1; 2: NT lower than cancer by 2; 3: NT lower than cancer by 3. The height of the bars (y-axis) is the number of patients. The sum height of red bars is much greater than the green bar, indicating that the IHC scores among the peritumoral stromal cells are much higher than the corresponding normal stromal cells.

To eliminate reading variation from individual labs, all stained TMAs were scanned at Johns Hopkins University using the Aperio imaging system, and the 20X scanned images were analyzed using the same computation tools to determine the overall staining intensity for each core. We further validated TMA interpretation by JHH pathologists for all TMAs to separate the stromal staining and epithelial staining. The pathologists’ readings used a four tier system based on the intensity and distribution: 0, undetectable; 1+, weak staining; 2+, medium staining, 3+, strong staining (Fig. 1A). In consistent with our previous observation, the periostin staining was observed mainly in peritumoral stromal cells and in some cases in tumor epithelial cells though the stronger staining was found in peritumoral stromal cells (Fig. 1A). The statistical analysis was then performed to answer the following questions: 1) whether periostin expression can differentiate prostate tissues including prostate cancer, non-tumor areas of prostate tissues, BPH, and PIN; 2) whether periostin expression is correlated with clinical variables, such as Gleason score.

To determine whether the expression of periostin could differentiate the CaP from non-tumor areas of prostate tissues, statistical analyses were performed for the three TMAs from JHU because these TMAs consist of both the prostate tumors and patient-matched non-tumorous prostate tissues. Periostin total staining and stromal staining were analyzed respectively. The periostin total staining was increased in prostate tumor with Gleason score 6 and up compared to paired non-tumorous prostate tissues (Fig. 1B). Since the periostin was mainly stained in stroma, histogram analysis was used to analyze the changes in IHC scores between periostin peritumoral stroma and paired non-tumorous stroma (Fig. 1C). The x-axis is the difference in IHC scores, which was calculated using the IHC score of periostin peritumoral stroma minus the score of paired non-tumorous stroma. The red bars indicate number of patients who had higher IHC scores in cancer compared to non-tumorous stroma, while the green bar indicates the lower IHC scores in cancer. The height of the bars (y-axis) is the number of patients. The sum height of red bars is significantly greater than the green bar, indicating that the IHC scores among the peritumoral stromal cells are significantly higher than the corresponding non-tumorous stromal cells. The Wilcoxon signed rank order test (paired, two-sided) gives the p-value for the significance of this difference (p = 2.75e-12).

**Fig 2:**
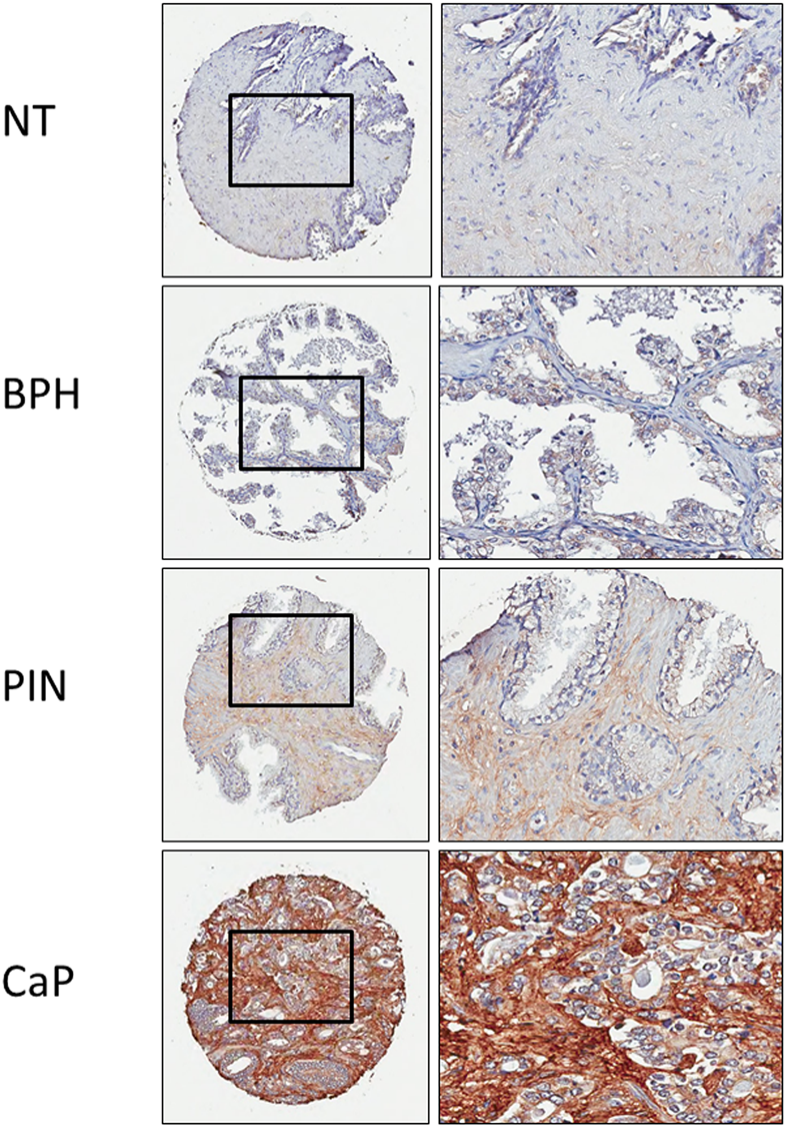
The periostin expression in different conditions of prostate diseases (TMAs from UCLA). The representative images of periostin expression in tissues of different prostate diseases. NT: patient paired non-tumorous prostate; BPH: benign prostatic hyperplasia, PIN: prostate intraepithelial neoplasias; and CaP: prostate adenocarcinoma.

To compare the periostin expression in prostate tumor and other conditions of prostate diseases, the TMAs from UCLA which consist of 197 cores of non-tumorous prostate (NT), 140 cores of benign prostatic hyperplasia (BPH), 64 cores of prostate intraepithelial neoplasia (PIN), and 963 cores of CaP were analyzed. Except the cores with tissue fall-off, 1233 cores were used in stromal staining analysis while 1247 cores were used in epithelial cell staining analysis. The periostin staining was detected with low background in non-tumorous prostate and BPH, and with increased staining in PIN, while most intensive staining was found in CaP (Fig. 2). Chi-squared test revealed that both periostin stromal staining and epithelial staining differentiated BPH from CaP including low grade CaP (Gleason score ≤6), with significant p-value of 2.2e-16 and 0.001, respectively (Tables 1 and 2, S1 Statistical Analysis). Periostin epithelial staining differentiated PIN from CaP including low grade CaP (Gleason score ≤6) (p = 0.001). In addition, periostin stromal staining differentiated low grade CaP (Gleason score ≤6) from high grade CaP (Gleason score ≤6) (p = 1.7e-05) (Tables 1 and 2, S1 Statistical Analysis). The results show that the periostin staining is positively correlated with the aggressiveness of prostate cancer.

**Table 1:** Periostin peritumoral stromal staining is positively associated with prostate adenocarcinoma (TMAs from UCLA).

		Periostin staining in stromal cells			Row total
		No (0)	Weak (1)	Medium & strong (2 &3)	
**Histology**	BPH *	55	39	36	130
	PIN	6	11	39	56
	CaP (GS≤6)	44	88	422	554
	CaP (GS≥7) *	9	25	278	312
**Column total**		114	163	775	1052

* p<0.01 compared to CaP (GS≤6)

**Table 2:** Periostin epithelial cell staining is positively associated with prostate adenocarcinoma (TMAs from UCLA).

		Periostin staining in epithelial cells			Row total
		No (0)	Weak (1)	Medium & strong (2 &3)	
**Histology**	BPH *	49	41	37	127
	PIN *	12	17	63	92
	CaP (GS≤6)	133	166	243	542
	CaP (GS≥7)	65	88	159	312
**Column total**		259	312	502	1073

* p<0.01 compared to CaP (GS≤6)

**Fig 3:**
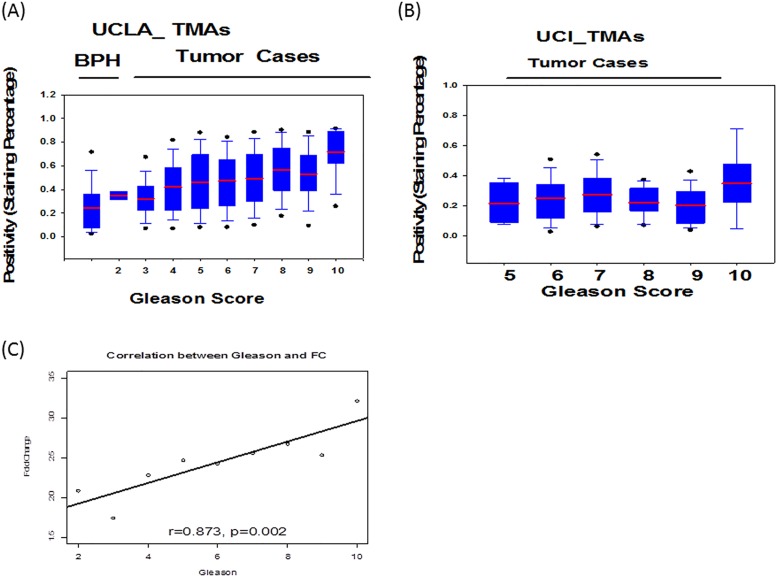
Correlation analysis of the positivity of total periostin staining and Gleason score. (A) Box plots of the positivity of periostin for the three TMAs from UCLA, (B) Box plots of the positivity of periostin for the three TMAs from UCI, (C) the positive correlation of fold changes and Gleason score using all nine TMAs. Percentage of staining (positivity) was calculated as total number of positive pixels divided by total number of pixels.

To determine the correlation of periostin expression and Gleason score, all nine TMAs were analyzed using the same image computational tool. Percentage of staining was calculated as total number of positive pixels divided by total number of pixels. The increases of periostin total staining intensity were found in the prostate tumors with increased Gleason score in the three TMAs from JHU (Fig. 1B), the three TMAs from UCLA (Fig. 3A), and the three TMAs from UCI (Fig. 3B). The results show that a positive correlation between total periostin staining and Gleason score (r = 0.87, p = 0.002) (Fig. 3C).

## Discussion

In the present study, we investigated the expression level of periostin in the primary prostate cancer tissues, non-tumorous prostate tissues, benign prostatic hyperplasia, and prostate intraepithelial neoplasia. This is the first study using such an expanded cohort to analyze periostin expression in prostate cancer and try to determine the clinical relevance of periostin elevation with the CaP aggressive phenotype.

High expression of periostin localized in either tumor epithelia or peritumoral stroma was observed consistently as previous reports [16–19]. Interestingly, while strong peritumoral periostin staining was observed in all TMAs, the periostin staining in tumor epithelia was only detected in the TMAs from UCLA and UCI, but not in the TMAs from JHU. This may have been caused by variations in patient cohorts and the technical pre-processing that includes sample collection, pathologist selection etc. during construction of the JHU TMAs. According to the statistical analysis, epithelial and stromal periostin expression was able to distinguish CaP form non-tumorous prostate, BPH and PIN (Tables 1 and 2, Figs 1 and 2, and S1 Statistical Analysis). In addition, the total periostin staining (epithelia and stromal) was shown to be correlated with increasing Gleason score (Fig. 3C). This indicates the peritumoral periostin staining was highly associated with the CaP aggressiveness.

Other studies have suggested that periostin expression is correlated with cancer metastasis and the bleak prognosis [9, 20, 21]. Li *et al* reported that over-expression of periostin was frequently observed in the stroma of nasopharyngeal carcinoma and matched lymph node metastases compared with the stroma of normal nasopharyngeal mucosa[16]. High stromal expression of periostin was also observed to be associated with shorter survival of prostate cancer [19]. However, notably the TMAs used in this study only consist of primary prostate tumors. It would be extremely useful to analyze the periostin expression in CaP recurrence cases that may progress to metastasis and death in the future.

In conclusion, although periostin was increased in both peritumoral stroma and epithelial CaP cells, periostin had major expression in the former cell type. Our data show that periostin expression is highly correlated with the CaP tumor aggressiveness, which supports our previous discovery. The results indicate that periostin has the potential to be used as a diagnostic tissue biomarker.

## Supporting Information

S1 Statistical Analysis The statistical analysis of periostin staining and status of prostate disease. (DOCX)

## References

[1] Tian Y, Bova GS, Zhang H. Quantitative glycoproteomic analysis of optimal cutting temperature-embedded frozen tissues identifying glycoproteins associated with aggressive prostate cancer. Anal Chem. 2011 9 15;83(18):7013–9. 10.1021/ac200815q 21780747 PMC4285776

[2] Ruan K, Bao S, Ouyang G. The multifaceted role of periostin in tumorigenesis. Cell Mol Life Sci. 2009 7;66(14):2219–30. 10.1007/s00018-009-0013-7 19308325 PMC11115806

[3] Kudo Y, Siriwardena BS, Hatano H, Ogawa I, Takata T. Periostin: novel diagnostic and therapeutic target for cancer. Histol Histopathol. 2007 10;22(10):1167–74. 17616943 10.14670/HH-22.1167

[4] Michaylira CZ, Wong GS, Miller CG, Gutierrez CM, Nakagawa H, Hammond R, et al Periostin, a cell adhesion molecule, facilitates invasion in the tumor microenvironment and annotates a novel tumor-invasive signature in esophageal cancer. Cancer Res. 2010 7 1;70(13):5281–92. 10.1158/0008-5472.CAN-10-0704 20516120 PMC3274349

[5] Erkan M, Kleeff J, Gorbachevski A, Reiser C, Mitkus T, Esposito I, et al Periostin creates a tumor-supportive microenvironment in the pancreas by sustaining fibrogenic stellate cell activity. Gastroenterology. 2007 4;132(4):1447–64. 17408641 10.1053/j.gastro.2007.01.031

[6] Xu D, Xu H, Ren Y, Liu C, Wang X, Zhang H, et al Cancer stem cell-related gene periostin: a novel prognostic marker for breast cancer. PLoS One. 2012;7(10):e46670 10.1371/journal.pone.0046670 23056395 PMC3467269

[7] Kikuchi Y, Kashima TG, Nishiyama T, Shimazu K, Morishita Y, Shimazaki M, et al Periostin is expressed in pericryptal fibroblasts and cancer-associated fibroblasts in the colon. J Histochem Cytochem. 2008 8;56(8):753–64. 10.1369/jhc.2008.951061 18443362 PMC2443605

[8] Morra L, Rechsteiner M, Casagrande S, von Teichman A, Schraml P, Moch H, et al Characterization of periostin isoform pattern in non-small cell lung cancer. Lung Cancer. 2011 5;76(2):183–90. 10.1016/j.lungcan.2011.10.013 22079858

[9] Zhu M, Fejzo MS, Anderson L, Dering J, Ginther C, Ramos L, et al Periostin promotes ovarian cancer angiogenesis and metastasis. Gynecol Oncol. 2010 11;119(2):337–44. 10.1016/j.ygyno.2010.07.008 20688362

[10] Tian Y, Yao Z, Roden RB, Zhang H. Identification of glycoproteins associated with different histological subtypes of ovarian tumors using quantitative glycoproteomics. Proteomics. 2011 12;11(24):4677–87. 10.1002/pmic.201000811 22113853 PMC3426283

[11] Tischler V, Fritzsche FR, Wild PJ, Stephan C, Seifert HH, Riener MO, et al Periostin is up-regulated in high grade and high stage prostate cancer. BMC Cancer. 2010;10:273 10.1186/1471-2407-10-273 20534149 PMC2903527

[12] Maresh EL, Mah V, Alavi M, Horvath S, Bagryanova L, Liebeskind ES, et al Differential expression of anterior gradient gene AGR2 in prostate cancer. BMC Cancer. [Research Support, N.I.H., Extramural]. 2010;10:680. 10.1186/1471-2407-10-680 PMC3009682 21144054

[13] Seligson DB, Hongo F, Huerta-Yepez S, Mizutani Y, Miki T, Yu H, et al Expression of X-linked inhibitor of apoptosis protein is a strong predictor of human prostate cancer recurrence. Clin Cancer Res. [Research Support, N.I.H., Extramural Research Support, Non-U.S. Gov't Research Support, U.S. Gov't, Non-P.H.S.]. 2007 10 15;13(20):6056–63. 10.1158/1078-0432.CCR-07-0960 17947468

[14] Krajewska M, Olson AH, Mercola D, Reed JC, Krajewski S. Claudin-1 immunohistochemistry for distinguishing malignant from benign epithelial lesions of prostate. Prostate. [Research Support, N.I.H., Extramural]. 2007 6 15;67(9):907–10. 17440968 10.1002/pros.20578

[15] Krajewska M, Kitada S, Winter JN, Variakojis D, Lichtenstein A, Zhai D, et al Bcl-B expression in human epithelial and nonepithelial malignancies. Clin Cancer Res. [Research Support, N.I.H., Extramural]. 2008 5 15;14(10):3011–21. 10.1158/1078-0432.CCR-07-1955 18483366 PMC4171052

[16] Li M, Li C, Li D, Xie Y, Shi J, Li G, et al Periostin, a stroma-associated protein, correlates with tumor invasiveness and progression in nasopharyngeal carcinoma. Clin Exp Metastasis. 2012 12;29(8):865–77. 10.1007/s10585-012-9465-5 22706927

[17] Wang H, Wang Y, Jiang C. Stromal protein periostin identified as a progression associated and prognostic biomarker in glioma via inducing an invasive and proliferative phenotype. Int J Oncol. 2013 5;42(5):1716–24. 10.3892/ijo.2013.1847 23467707

[18] Gunia S, Jain A, Koch S, Denzinger S, Gotz S, Niessl N, et al Periostin expression correlates with pT-stage, grading and tumour size, and independently predicts cancer-specific survival in surgically treated penile squamous cell carcinomas. J Clin Pathol. 2013 4;66(4):297–301. 10.1136/jclinpath-2012-201262 23372176

[19] Nuzzo PV, Rubagotti A, Zinoli L, Ricci F, Salvi S, Boccardo S, et al Prognostic value of stromal and epithelial periostin expression in human prostate cancer: correlation with clinical pathological features and the risk of biochemical relapse or death. BMC Cancer.12:625 10.1186/1471-2407-12-625 23273263 PMC3553030

[20] Wu G, Wang X, Zhang X. Clinical implications of periostin in the liver metastasis of colorectal cancer. Cancer Biother Radiopharm. 2013 5;28(4):298–302. 10.1089/cbr.2012.1374 23347152

[21] Kyutoku M, Taniyama Y, Katsuragi N, Shimizu H, Kunugiza Y, Iekushi K, et al Role of periostin in cancer progression and metastasis: inhibition of breast cancer progression and metastasis by anti-periostin antibody in a murine model. Int J Mol Med. 2011 8;28(2):181–6. 10.3892/ijmm.2011.712 21617848

