# Correction: Overexpression of Periostin in Stroma Positively Associated with Aggressive Prostate Cancer

**DOI:** 10.1371/journal.pone.0130333

**Published:** 2015-06-04

**Authors:** Yuan Tian, Caitlin H. Choi, Qing Kay Li, Farah B. Rahmatpanah, Xin Chen, Sara Ruth Kim, Robert Veltri, David Chia, Zhen Zhang, Dan Mercola, Hui Zhang

There are errors in the Funding section. The correct funding information is as follows: This work was supported by National Institutes of Health, National Cancer Institute, the Early Detection Research Network (EDRN, U01CA152813) to HZ, the Prostate Cancer Biorepository Network, Department of Defense Grant Number W81XWH-10-2-0056, and Early Detection Research Network NCI CA86366 to DC. The funders had no role in study design, data collection and analysis, decision to publish, or preparation of the manuscript.

## References

[1] Tian Y, Choi CH, Li QK, Rahmatpanah FB, Chen X, Kim SR, et al (2015) Overexpression of Periostin in Stroma Positively Associated with Aggressive Prostate Cancer. PLoS ONE 10(3): e0121502 doi:10.1371/journal.pone.0121502 25781169 10.1371/journal.pone.0121502 PMC4362940

