# Supplementary material for: Overexpression of Periostin in Stroma Positively Associated with Aggressive Prostate Cancer
Source: PLoS One. 2015 Mar 17;10(3):e0121502. doi: 10.1371/journal.pone.0121502 (PMC4362940; doi:10.1371/journal.pone.0121502)
Supplement: S1 Statistical Analysis — (DOCX) [file pone.0121502.s001.docx]

**Statistical analysis for periostin staining and status of prostate disease**

**I. For table 1: periostin stromal staining**

**Test 1**. **BPH vs CaP**

|  | **No (0)** | **Weak (1)** | **Medium & strong (2 &3)** |
| --- | --- | --- | --- |
| BPH | 55 | 39 | 36 |
| CaP | 53 | 113 | 700 |

Pearson's Chi-squared test:

X-squared = 200.9836, df = 2, p-value < 2.2e-16

**Test 2.**  **PIN vs CaP**

|  | **No (0)** | **Weak (1)** | **Medium & strong (2 &3)** |
| --- | --- | --- | --- |
| PIN | 6 | 11 | 39 |
| CaP | 53 | 113 | 700 |

Pearson's Chi-squared test

X-squared = 4.2571, df = 2, p-value = 0.119

Fisher's Exact Test for Count Data

p-value = 0.1023

**Test 3.**  **BPH vs CaP (GS<=6)**

|  | **No (0)** | **Weak (1)** | **Medium & strong (2 &3)** |
| --- | --- | --- | --- |
| BPH | 55 | 39 | 36 |
| CaP (GS<=6) | 44 | 88 | 422 |

Pearson's Chi-squared test

X-squared = 134.1726, df = 2, p-value < 2.2e-16

**Test 4.**  **PIN vs CaP (GS<=6)**

|  | **No (0)** | **Weak (1)** | **Medium & strong (2 &3)** |
| --- | --- | --- | --- |
| PIN | 6 | 11 | 39 |
| CaP (GS<=6) | 44 | 88 | 422 |

Pearson's Chi-squared test

X-squared = 1.2064, df = 2, p-value = 0.547

Fisher's Exact Test for Count Data

p-value = 0.5042

**Test 5.**  **CaP (GS<=6) vs CaP (GS>6)**

|  | **No (0)** | **Weak (1)** | **Medium & strong (2 &3)** |
| --- | --- | --- | --- |
| CaP (GS<=6) | 44 | 88 | 422 |
| CaP (GS>=7) | 9 | 25 | 278 |

Pearson's Chi-squared test

X-squared = 21.948, df = 2, p-value = 1.714e-05

Fisher's Exact Test for Count Data

p-value = 9.683e-06

**II. For table 2: periostin epithelial staining**

**Test 1**. **BPH vs CaP**

|  | **No (0)** | **Weak (1)** | **Medium & strong (2 &3)** |
| --- | --- | --- | --- |
| BPH | 49 | 41 | 37 |
| CaP | 198 | 254 | 402 |

Pearson's Chi-squared test

X-squared = 18.5982, df = 2, p-value = 9.151e-05

**Test 2.**  **PIN vs CaP**

|  | **No (0)** | **Weak (1)** | **Medium & strong (2 &3)** |
| --- | --- | --- | --- |
| PIN | 12 | 17 | 63 |
| CaP | 198 | 254 | 402 |

Pearson's Chi-squared test

X-squared = 15.2684, df = 2, p-value = 0.0004836

Fisher's Exact Test for Count Data

p-value = 0.0005567

**Test 3**. **BPH vs CaP (GS<=6)**

|  | **No (0)** | **Weak (1)** | **Medium & strong (2 &3)** |
| --- | --- | --- | --- |
| BPH | 49 | 41 | 37 |
| CaP (GS<=6) | 133 | 166 | 243 |

Pearson's Chi-squared test

X-squared = 13.6104, df = 2, p-value = 0.001108

**Test4. PIN vs CaP (GS<=6)**

|  | **No (0)** | **Weak (1)** | **Medium & strong (2 &3)** |
| --- | --- | --- | --- |
| PIN | 12 | 17 | 63 |
| CaP (GS<=6) | 133 | 166 | 243 |

Pearson's Chi-squared test

X-squared = 17.676, df = 2, p-value = 0.0001451

Fisher's Exact Test for Count Data

p-value = 0.0001567

**Test 5. CaP (GS<=6) vs CaP (GS>6)**

|  | **No (0)** | **Weak (1)** | **Medium & strong (2 &3)** |
| --- | --- | --- | --- |
| CaP (GS<=6) | 133 | 166 | 243 |
| CaP (GS>=7) | 65 | 88 | 159 |

Pearson's Chi-squared test

X-squared = 3.1427, df = 2, p-value = 0.2078

Fisher's Exact Test for Count Data

p-value = 0.2085
